# Development and Validation of an Extracellular Matrix Gene Expression Signature for Prognostic Prediction in Patients with Uveal Melanoma

**DOI:** 10.3390/ijms26094317

**Published:** 2025-05-01

**Authors:** Alejandro Mejía-García, Carlos A. Orozco, Julius Herzog, Oscar Alarcón-Betancourth, Alexandra Meneses-Torres, Marcela Ramírez, Johanna González, Yina Zambrano, Alba Lucia Combita, Diego A. Bonilla, Seth Frietze

**Affiliations:** 1 Department of Human Genetics, McGill University, Montreal, QC H3A 0G4, Canada; alejandro.mejiagarcia@mail.mcgill.ca; 2 Cancer Biology Research Group, Instituto Nacional de Cancerología, Bogotá 110311, Colombia; corozco@cancer.gov.co (C.A.O.); 3 Translational Research Group in Oncology, Instituto Nacional de Cancerología, Bogotá 110311, Colombia; 4 Department of Biomedical and Health Sciences, University of Vermont, 106 Carrigan Drive, 302 Rowell, Burlington, VT 04505, USA; 5 Master Program in Epidemiology, Fundación Universitaria del Área Andina, Bogotá 110311, Colombia; ameneses17@estudiantes.areandina.edu.co (A.M.-T.); 6 Health and Sport Sciences Research Group, School of Health and Sport Sciences, Fundación Universitaria del Área Andina, Bogotá 110311, Colombia; 7 Department of Microbiology, Universidad Nacional de Colombia, Bogotá 111311, Colombia; 8 Research Division, Dynamical Business & Science Society, DBSS International SAS, Bogotá 110311, Colombia; dabonilla@dbss.pro; 9 Hologenomiks Research Group, Department of Genetics, Physical Anthropology and Animal Physiology, University of the Basque Country (UPV/EHU), 48940 Leioa, Spain

**Keywords:** uveal melanoma, extracellular matrix, prognostic signature, gene expression profiling, tumor microenvironment, epithelial–mesenchymal transition

## Abstract

Uveal melanoma (UVM) is an aggressive cancer with a poor prognosis, particularly in metastatic cases. This study aimed to develop and validate a novel extracellular matrix (ECM) gene expression signature to predict prognosis and stratify patients by risk. ECM-related genes were identified and used to construct a prognostic model through Lasso–Cox regression analysis, leveraging RNA sequencing data from 80 UVM patients in The Cancer Genome Atlas (TCGA). The model was validated using an independent cohort of 63 UVM patients. Survival analyses, immune infiltration profiling, and functional enrichment analyses were conducted to evaluate the biological significance and clinical utility of the signature. The ECM signature stratified patients into high- and low-risk groups with significant differences in survival outcomes. High-risk patients showed elevated expression of *MMP1* and *MMP12*, which are associated with ECM remodeling and immune modulation, alongside increased infiltration of immunosuppressive cells, such as M2 macrophages. Validation confirmed the prognostic value of the signature across cohorts. Functional analyses highlighted the involvement of ECM-related pathways, epithelial–mesenchymal transition, and immune system interactions in tumor progression. This ECM gene expression signature is a robust prognostic tool for UVM, offering insights into tumor biology and immune microenvironment interactions. It holds promise for improving patient stratification and guiding personalized therapeutic strategies. Further research is warranted to explore the functional roles of these genes in UVM progression.

## 1. Introduction

Uveal melanoma (UVM) is the predominant intraocular cancer in the United States, with an incidence of approximately 5.1 cases per million individuals, accounting for 83% of all ocular melanomas. The prevalence of UVM is notably higher among individuals of European descent compared to those of Asian and African ancestry, with incidence rates ranging from 1.3 to 8.6 cases per million in Europe, in contrast to 0.2 to 0.3 cases per million in Asia and Africa [1]. Despite its rarity, UVM carries a high mortality rate, with a global 5-year survival rate of approximately 70%. However, this rate is significantly lower in high-risk regions such as Finland, where survival rates drop to around 62%, largely due to late-stage diagnoses and the aggressive nature of the disease [1,2]. While primary tumor diagnosis is prevalent among UVM patients, approximately 50% of cases advance to metastasis, with a median overall survival post-metastasis ranging between 6 and 12 months [3]. Currently, standard treatment modalities for primary tumors encompass enucleation, surgical excision, irradiation, and radiotherapy, with selection based on tumor size. However, in cases of metastasis, therapeutic options such as chemotherapy, immunotherapy, and molecular-targeted therapy have demonstrated limited efficacy [4]. The imperative for developing novel targeted therapies capable of extending survival rates among metastatic patients is needed.

Furthermore, UVM tumors display considerable heterogeneity, characterized by various chromosomal aberrations, notably monosomy of chromosome 3, which is associated with an increased risk of metastasis [5,6]. Additional genomic alterations involve the loss of chromosomes 1p and 6q, coupled with gains in 6p and 8 [7]. Notably, evidence suggests that these genomic changes may occur progressively, complicating prognostic evaluations [8]. As a result, prognostic signatures leveraging gene expression have emerged as promising tools for patient stratification and metastasis prediction [9,10,11,12,13,14,15,16,17]. Moreover, the design of such signatures enables the investigation of critical biological processes implicated in UVM development and progression, including UV–light responses and pyroptosis [9,18].

The extracellular matrix (ECM) acts as a pivotal regulator of metastasis, facilitating the migration and invasion of tumor cells into adjacent tissues and distant organs [19]. It is worth noting that, in the context of UVM, dysregulation of ECM-related proteins, such as matrix metalloproteinases (MMPs), play a central role in ECM remodeling, thereby promoting invasion [20]. ECM proteins have been implicated in metastasis and are associated with poorer survival outcomes in UVM patients [21]. Considering the crucial role of ECM remodeling in metastasis and its significant impact on patient survival, the development of prognostic signatures incorporating these key biological processes holds great potential for improving survival prediction and guiding personalized treatment strategies.

Hence, this study introduces a novel gene expression signature aimed at predicting the prognosis of patients diagnosed with UVM. Our investigation extends to exploring the expression patterns of specific extracellular matrix-associated genes and their correlation with the observed biological features in individuals with UVM. The findings of our research might offer new insights into the diagnosis, prognosis, and biological implications of ECM proteins in the progression of UVM.

## 2. Results

### 2.1. Clinicopathological Features of the Study Population

A summary of the clinicopathological characteristics of the study cohort is presented in Supplementary Table S1. Overall, males comprised 56.25% of the patients, while females accounted for 43.75%, with the mean age of diagnosis being 61 years. The majority of patients underwent diagnosis via enucleation (97.5%), predominantly presenting with stage IIIA disease (38.75%). The most prevalent cytogenetic anomaly observed was the loss of chromosome 3 or gain of chromosome 8q (22.5%), followed by the gain of chromosome 6p (13.75%). On average, patients were followed for up to 787 days, with a mean duration of 608 days from diagnosis to the occurrence of a new tumor event.

### 2.2. Risk Model Construction

**Figure 1:**
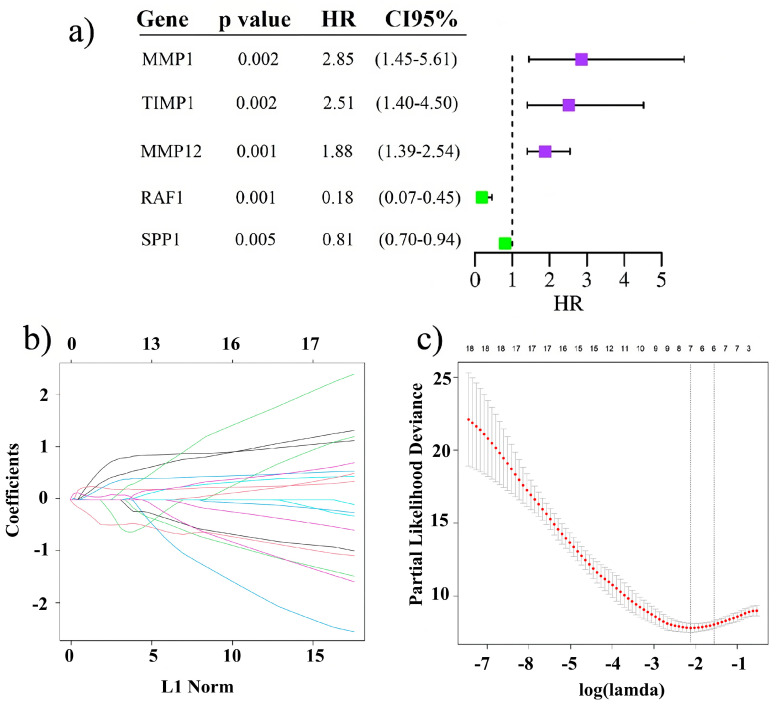
Construction of the prognostic model for Uveal Melanoma (UVM) patients. (**a**) Forest plot displaying the prognostic signature with the hazard Ratio (HR) and a 95% confidence interval. (**b**) Plot of coefficient pathways, with each curve corresponding to a gene. The X-axis shows the number of non-zero coefficients at the current lambda, and the Y-axis shows the value of the coefficient for that gene at each lambda. (**c**) Cross-validation (CV) is employed to select the optimal lambda value for the Lasso–Cox model. The X-axis of the plot displays different Lambda values, while the Y-axis represents the CV error. The point at which the CV–error curve reaches its minimum is indicated by the left vertical line.

To determine the prognostic signature of the extracellular matrix in UVM, we conducted a comprehensive analysis and explored the role of ECM-associated genes on the prognosis of UVM patients. Univariate Cox regression was performed using a comprehensive list of 97 ECM genes, identifying 43 that significantly influenced overall survival in the UVM cohort from The Cancer Genome Atlas (TCGA) (Supplementary Table S2). We then applied Lasso–Cox regression with L1 regularization that allows for feature selection to distill this set further. This not only reduced the gene set to the five most critical predictors (Figure 1a) but also balanced the prognostic model’s complexity against its predictive power, as evidenced by the coefficient paths (Figure 1b). The optimal lambda value for the regression was determined using 5-fold cross-validation (Figure 1c).

### 2.3. Survival Analysis in the Training and Validation Cohorts

**Figure 2:**
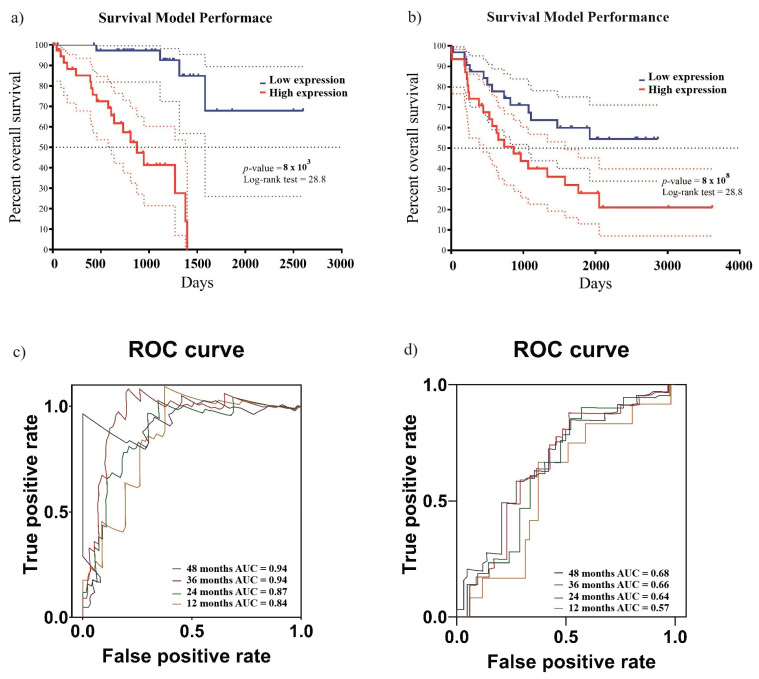
Survival analysis based on the prognostic model. To evaluate the efficacy of the risk model, survival analyses were conducted distinguishing between the high-risk group and the low-risk group. (**a**) Demonstrates the survival analysis for the TCGA cohort; (**b**) shows the analysis for the validation cohort. The ROC curves shown in (**c**,**d**) depict the model’s accuracy in predicting patients’ overall survival over different yearly intervals in the training cohort and the validation cohort, respectively.

To assess the prognostic value of the five-gene expression signature, we calculated a risk score for each of the 80 UVM patients. as described in the Methods Section. The robustness of the risk model was then assessed using survival analysis, comparing the outcomes between the high-risk and low-risk groups within both the training cohort (Figure 2a) and the validation cohort (Figure 2b). The Kaplan–Meier curves demonstrate a clear correlation between risk scores and survival outcomes, with high-risk scores associated with a poorer prognosis and low-risk scores with better survival (Figure 2a,b). Receiver operating characteristic (ROC) curves, depicting the survival prediction capability at 1, 2, 3, and 4 years, yielded areas under the curve (AUC) of 0.84, 0.87, 0.94, and 0.94 in the training cohort (Figure 2c) and 0.57, 0.64, 0.66, and 0.68 in the validation cohort (Figure 2d), respectively.

### 2.4. Correlation Analysis

To explore differences in clinicopathological characteristics across the risk groups defined by our prognostic model, we performed a correlation analysis relating these characteristics to patient risk scores. Phenotypic data from the database were examined for associations with high- or low-gene-expression groups, using chi-square exact tests to assess statistical significance (Table 1). Our analysis indicated that the high-risk group had a higher frequency of distant metastases, particularly to the liver, and was more likely to have a positive tumor status at the end of the follow-up period (*p* < 0.05). Other clinicopathological variables did not show notable differences between the two groups (Supplementary Table S3). Additionally, we assessed the relationship between the expression of specific genes in our model and the clinicopathological parameters within the TCGA cohort. This analysis demonstrated that expression levels of *MMP1* and *MMP12* were increased in patients with residual tumors post-treatment, whereas *RAF1* expression was higher in patients who were tumor-free post-treatment (Supplementary Figure S1).

### 2.5. Differential Expression Analysis and Gene Enrichment Analysis

**Figure 3:**
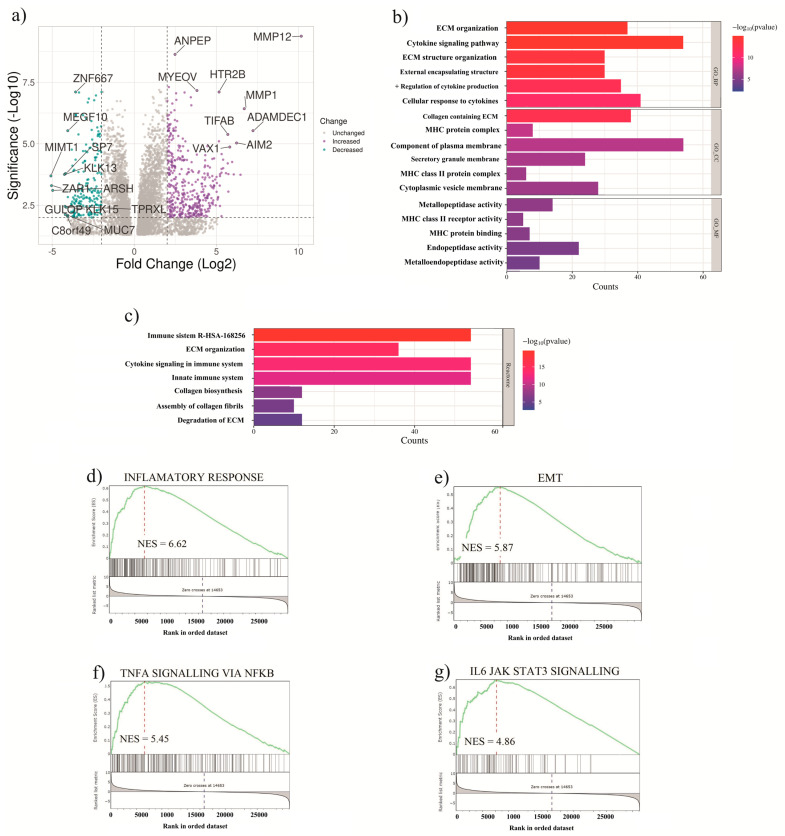
Differential expression analysis and gene enrichment analysis. (**a**) Volcano plot displaying differentially expressed genes (DEGs) between the low- and high-risk groups. (**b**) Results of the functional enrichment analysis, including gene ontology (GO) annotations for biological processes, molecular functions, and cellular components. (**c**) Pathway analysis (REACTOME) highlighting the most significantly enriched pathways associated with the differentially expressed genes. (**d**–**g**) Gene set enrichment analysis (GSEA) depicting the enrichment of pathways associated with inflammatory responses (FDR < 0.001), epithelial-to-mesenchymal transition (EMT) (FDR = 0.001), TNFα signaling via NFκB (FDR = 0.004), and IL-6 JAK-STAT3 signaling (FDR = 0.007).

A differential gene expression analysis was conducted to compare patients with low risk scores to those with high risk scores. Our results indicated the upregulation of genes associated with the ECM, such as *MMP1* and *MMP12*, as well as immune system-related genes, in the high-risk group (Figure 3a). The expression levels of *MMP1*, *MMP12*, and *RAF1* among UVM patients from the TCGA cohort, distinguishing between those with or without tumors post-treatment, are shown in Supplementary Figure S1. In contrast, the expression of *RAF1* was significantly lower in patients with tumors. These findings underscore the potential role of *MMP1*, *MMP12*, and *RAF1* as noteworthy indicators of treatment response and tumor presence in UVM patients.

On the other hand, GO enrichment analysis was employed to elucidate the differentially regulated pathways between high-risk and low-risk patients. Notably, our findings revealed the enrichment of several biological processes linked to cancer, including cellular functions associated with ECM proteins and immune functions, potentially contributing to the poorer prognosis observed in these patients (Figure 3b,c). Subsequently, the GSEA was performed using the same patient groups, revealing the enrichment of pathways associated with the inflammatory response, epithelial-mesenchymal transition (EMT), TNFα signaling via NFκB, and IL6 JAK-STAT signaling in the high-risk group (Figure 3d–g). These findings contribute to the molecular mechanisms underlying the aggressive nature of high-risk tumors, emphasizing the involvement of inflammatory and immune-related pathways in disease progression.

### 2.6. Drug Sensitivity

**Figure 4:**
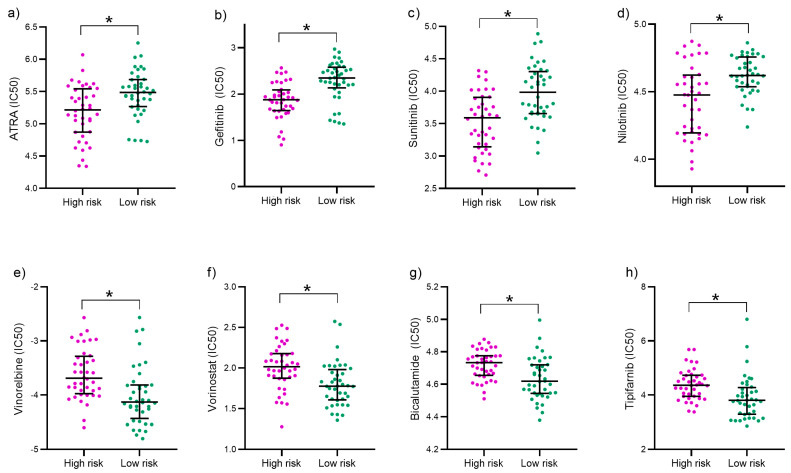
Analysis of antitumor drug sensitivity: dispersion plots reveal distinct IC50 sensitivities for various drugs across risk groups. In the low-risk group, differential sensitivities are observed for ATRA (**a**), Gefitinib (**b**), Sunitinib (**c**), and Nilotinib (**d**), while in the high-risk group, sensitivities vary for Vinorelbine (**e**), Vorinostat (**f**), Bicalutamide (**g**), and Tipifarnib (**h**). To assess statistical significance, *p*-values from the Mann–Whitney U test are presented (* *p* <0.001), indicating significant differences between groups. IC50 values are expressed as the negative logarithm of the micromolar concentration.

To identify possible therapeutic options that could potentially enhance survival in the high-risk category, we conducted a drug sensitivity analysis using the “pRRophetic” package in R. This analysis identified several drugs, including ATRA, Gefitinib, and Nilotinib, which demonstrated lower half-maximal inhibitory concentration (IC50) values in high-risk patients (*p* < 0.05), suggesting a greater sensitivity to these treatments. Conversely, a different set of anticancer drugs showed increased efficacy in the low-risk group, as indicated by their IC50 values (Figure 4). These results provide insights into tailored therapeutic strategies that may improve outcomes for patients based on their risk stratification.

### 2.7. Estimation of Immune Infiltration

**Figure 5:**
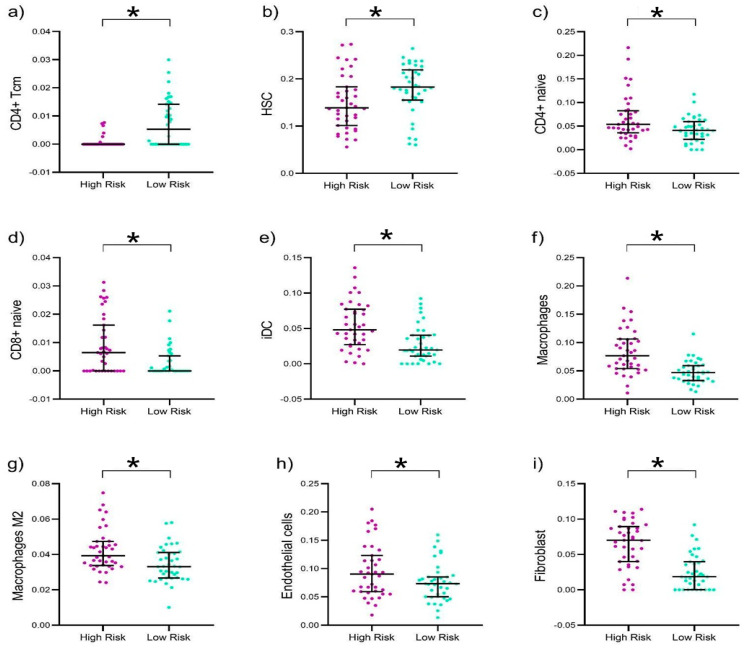
Evaluation of immune and stromal cell infiltration in the UVM TCGA cohort. Elevated infiltration of CD4 tissue central memory cells (CD4+ Tcm) (**a**) and hematopoietic stem cells (HSCs) (**b**) was observed in the low-risk UVM patients. In contrast, higher levels of CD4+ naive cells (**c**), CD8+ naive cells (**d**), immature dendritic cells (iDC) (**e**), macrophages (**f**), macrophages M2 (**g**), endothelial cells (**h**), and fibroblasts (**i**) were detected in the high-risk group. Dispersion plots are presented, illustrating the median and interquartile range, with *p*-values corresponding to the Mann–Whitney U test (* *p* < 0.001).

Using the xCell deconvolution tool in R, we analyzed bulk RNA sequencing data from TCGA UVM patients to estimate the infiltration levels of 64 immune and stromal cell types. Our analysis identified distinct infiltration patterns between the risk groups (Figure 5). Patients in the high-risk group showed increased infiltration of fibroblasts, M2 macrophages, and other cells with immunosuppressive profiles, which could explain their less favorable clinical outcomes. In contrast, the low-risk group’s tumors were more enriched with hematopoietic stem cells (HSCs) and central memory CD4 T-cells (CD4+ Tcm), indicating a potentially more robust immune response against the tumor.

**Figure 6:**
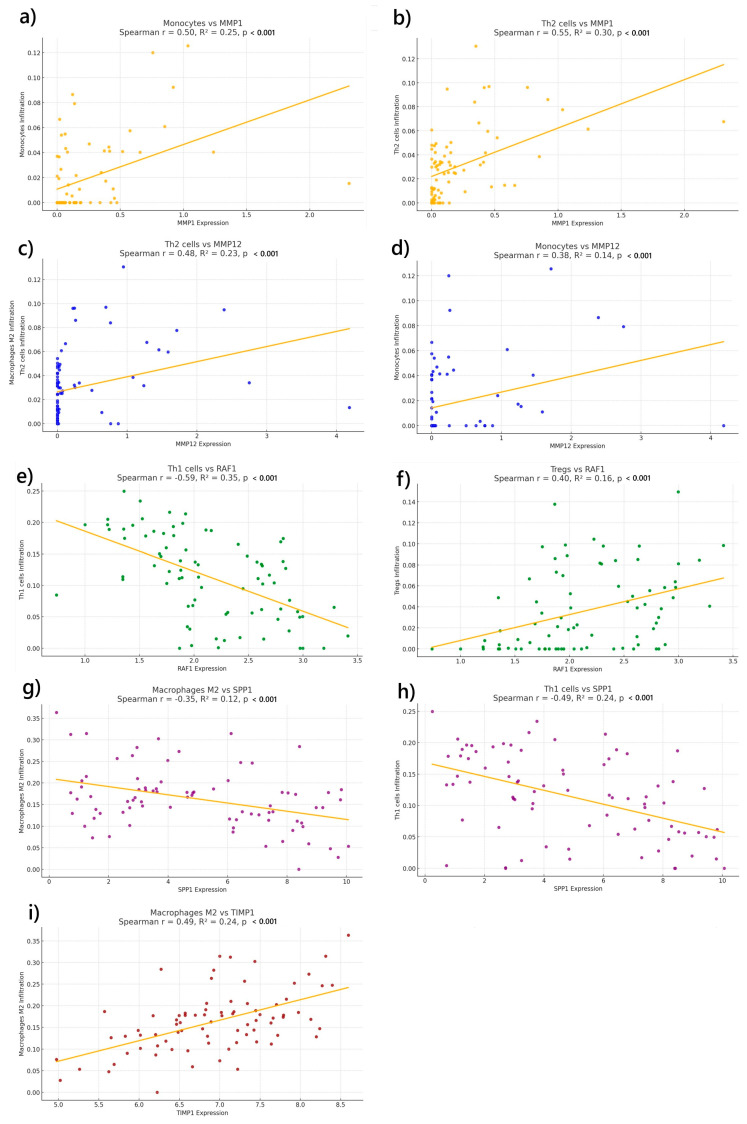
Correlation analysis between the expression of ECM signature genes and immune cell infiltration. Scatter plots show Spearman correlations between the expression levels of five ECM-related genes (*MMP1*, *MMP12*, *RAF1*, *SPP1*, and *TIMP1*) and the estimated infiltration of selected immune cell types in TCGA UVM samples. Each plot includes the regression line, correlation coefficient (r), R^2^, and *p*-value. Panels represent the following gene–cell type pairs: (**a**) *MMP1* vs. monocytes, (**b**) *MMP1* vs. Th2 cells, (**c**) *MMP12* vs. Th2, (**d**) *MMP12* vs. monocytes, (**e**) *RAF1* vs. Th1 cells, (**f**) *RAF1* vs. Tregs, (**g**) *SPP1* vs. M2 macrophages, (**h**) *SPP1* vs. Th1 cells, and (**i**) *TIMP1* vs. M2 macrophages.

To further explore the relationship between the ECM signature and the immune microenvironment, we conducted a correlation analysis comparing the expression levels of the five-gene ECM expression signature (*MMP1*, *MMP12*, *RAF1*, *SPP1*, and *TIMP1*) with immune cell infiltration patterns. Specifically, we examined both immunosuppressive cell populations (M2 macrophages, monocytes, Th2 cells, and T regulatory cells [Tregs]) and immune-effector T cells (Th1). As shown in Figure 6, *MMP1*, *MMP12*, and *TIMP1* expression showed significant positive correlations with immunosuppressive cell types, particularly monocytes, Th2 cells, and M2 macrophages. In contrast, the expression levels of *RAF1* and *SPP1* (which were associated with a protective effect in our prognostic model) exhibited negative correlations with Th1 cell infiltration. These findings suggest that their favorable prognostic role may not stem from Th1-mediated immune responses but rather from alternative mechanisms involving tumor progression or ECM remodeling.

### 2.8. Our Prognostic Signature in a UVM Single-Cell RNA-Seq Cohort 

**Figure 7:**
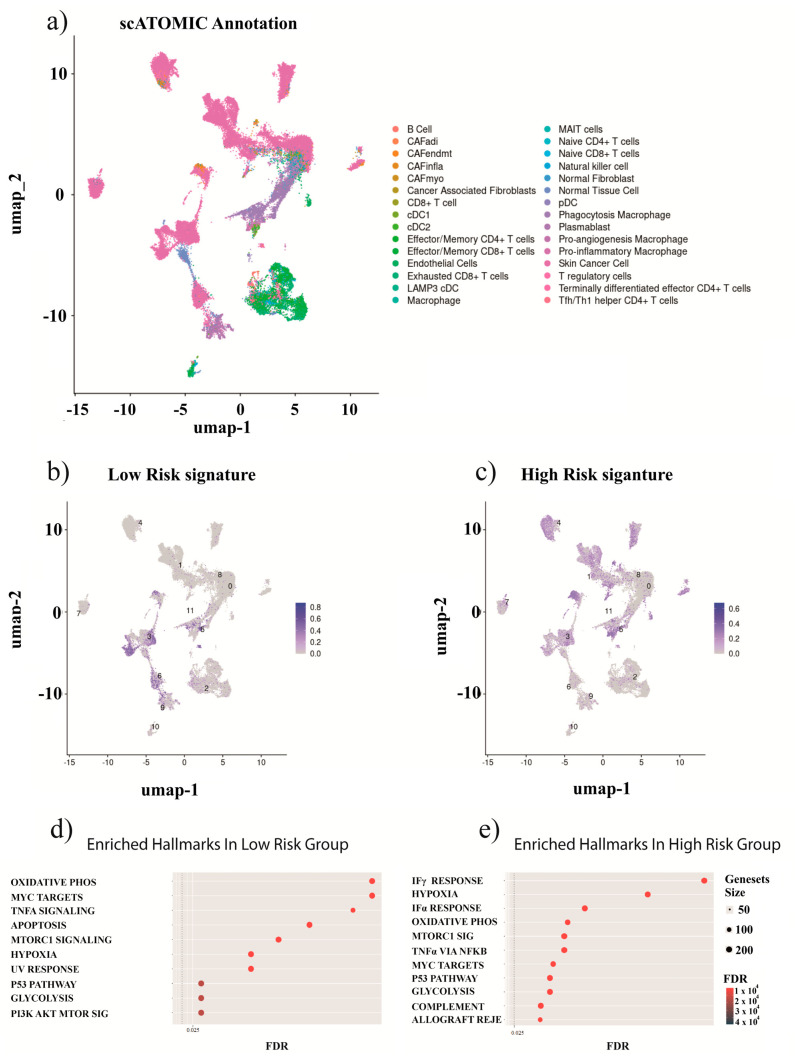
Single-cell analysis of the UVM prognostic gene signature. In panel (**a**), the annotated subpopulations identified within the UVM single-cell dataset are depicted. Panels (**b**,**c**) depict the expression patterns of genes constituting the low-risk and high-risk signatures across individual cells, highlighting their distinct distributions. Cluster numbers represent unsupervised groupings from the analysis pipeline and are included for reference only. The functional enrichment analysis provided in panels (**d**,**e**) demonstrates the biological processes and pathways that are differentially activated in cells expressing the low-risk and high-risk gene signatures, respectively.

We further investigated our prognostic signature in a single-cell RNA sequencing (scRNA-seq) dataset derived from metastatic UVM tumors (GSE139829). These results confirm the expression patterns of genes implicated in the high-risk prognostic signature and demonstrate their distribution across different cell types within the tumor microenvironment. The annotated cell types across the tumor samples (103,703 cells) include the presence of cancer cells, T cells, B cells, monocytes, and NK cells (Figure 7a). The expression patterns of genes associated with high-risk signatures, namely *MMP1*, *MMP12*, and *TIMP1*, indicate their presence in a subset of macrophages, endothelial cells, and cancer-associated fibroblasts (CAFs) (Figure 7b and Supplementary Figure S2b). In contrast, elevated *SPP1* expression was detected in macrophages and CAFs (Supplementary Figure S2a), and high levels of *RAF1* expression were found in T-reg cells, NK cells, naive T-CD8 cells, and CAFs (Supplementary Figure S2a). These patterns of expression were found in both primary and metastatic tumors (Supplementary Figure S3). Furthermore, we observed the upregulation of various cancer-associated hallmarks in cells expressing these high-risk genes, including TNF-alpha, UV response, angiogenesis, *MYC,* and *KRAS* (Figure 7e). Finally, we observed an upregulation of TNF-alpha signaling, MTORC1, and apoptosis in cells expressing the low-risk genes (Figure 7d).

### 2.9. Association Between the ECM Signature and Uveal Melanoma Molecular Subtypes

We evaluated the association between the ECM signature risk score and various molecular subtypes of uveal melanoma by comparing the risk scores across different subtype clusters. Our analysis revealed a significant association between higher ECM risk scores and SCNA clusters 3 and 4 when compared to cluster 1 (Supplementary Figure S4 and Supplementary Table S4). Additionally, a notable difference in ECM risk scores was observed between SCNA clusters 3 and 4. In the context of DNA methylation clusters, we identified a significant difference between cluster 4 and the other clusters, with cluster 4 exhibiting higher ECM risk scores. Similarly, significant associations were found for both mRNA and lncRNA clusters, where clusters 3 and 4 demonstrated elevated ECM risk scores compared to cluster 1.

## 3. Discussion

The extracellular matrix is critical to the maintenance of normal tissue homeostasis in the eye, contributing to corneal transparency, vitreous hydration, intraocular pressure regulation, and angiogenesis [22]. Remodeling of the ECM is mediated by dynamic interactions with regulatory molecules such as TGF-β2, which modulate its composition and function [22]. However, aberrant changes in ECM structure can significantly influence tumor behavior, facilitating cancer cell migration and invasion via basement membrane interactions and promoting cell proliferation and angiogenesis through altered integrin signaling and growth factor release [19]. The dysregulation of matrix metalloproteinases (MMPs) can significantly influence the onset and progression of cancer by disrupting the balance of ECM remodeling [23]. In the specific context of UVM, there is aberrant upregulation of several MMPs, including *MMP1*, *MMP9*, *MMP10*, *MMP11*, *MMP13*, and *MMP14*, compared to normal uveal tissue [21]. This aberrant expression pattern highlights the complex role of ECM components in UVM pathogenesis.

Given the established importance of the ECM, our study advances the understanding of its perturbation in uveal melanoma. We identify a prognostic signature that not only delineates patients into distinct risk categories but also implicates specific ECM genes as key mediators of aggressive disease phenotypes. Our model effectively stratified patients into high- and low-risk groups, with high-risk individuals exhibiting significantly shorter overall survival and metastasis-free survival (Figure 2). Notably, two of the five genes integrated into our model encode the matrix metalloproteinases *MMP1* and *MMP12*, both of which were associated with an elevated risk of poor survival. *MMP1*, identified as upregulated in cancers such as human oral squamous-cell carcinoma and cervical squamous-cell carcinoma [24,25], plays a pivotal role in tumor growth and cell motility contributes to tumor progression through extracellular-matrix remodeling and modulation of immune-cell interactions [26]. Recent single-cell and spatial transcriptomic analyses have shown that MMP1-expressing malignant subsets are associated with enhanced metastatic potential and immunosuppressive microenvironments [26]. This mechanism involves *MMP1* 3′UTR interacting with the microRNA miR-188-5p, a known tumor suppressor [26]. Additionally, MMP1 facilitates cancer progression by directly remodeling the ECM, targeting numerous ECM components for proteolysis. This process promotes migration, invasion, and EMT and inhibits apoptosis [27].

On the other hand, *MMP12* (encoding macrophage metalloelastase) is predominantly expressed in macrophages, immature dendritic cells (iDCs), and corneal epithelium. *MMP12* expression stimulates neutrophil recruitment, increasing cell migration and proliferation [28]. MMP12 degrades several ECM structures, including IV collagen, fibronectin, heparan sulfate proteoglycans, and fibronectin. Importantly, MMP12 can degrade the basement membrane, which is critical for promoting macrophage invasion into tissues [29]. Due to ECM-associated activities, MMP12 has an important role in inflammatory diseases such as colitis [30], asthma, fibrosis, chronic obstructive pulmonary disease (COPD) [31], and cardiovascular disease [32]. Within the context of uveal melanoma, *MMP12* expression has been previously reported as a negative prognostic marker, identified as a constituent of an immunological prognosis signature for UVM [33].

In our model, *TIMP1* emerged as a gene associated with poor survival (Figure 1a). *TIMP1*, a tissue inhibitor of matrix metalloproteinases (TIMMPs), is recognized for its potential to hinder tumor process [34]. Paradoxically, elevated *TIMP1* expression has been consistently linked to unfavorable prognoses across various cancers, including breast, lung, endometrial, pancreatic, and skin cancers, including melanoma and UVM [35,36,37,38,39,40,41]. In the latter, a co-expression study revealed a significant association between *TIMP1* expression and metastasis, suggesting that its elevated levels might represent a physiological attempt to regulate MMPs and control the tumor, albeit unsuccessfully [42]. Beyond MMP inhibition, *TIMP1*’s roles extend to the activation of the PI3K signaling pathway by interacting with PDK1, thereby generating survival signals and promoting proliferation in melanocytes [35]. Furthermore, TIMP1 has been implicated in the accumulation of CAFs and stimulation of their proliferation via the ERK1/2 pathway [43]. CAFs, in turn, enhance the invasiveness of melanoma cells by secreting growth factors, cytokines, and chemokines, thereby fostering a proliferative tumor microenvironment [44].

In our study, two genes, *RAF1* and *SPP1*, are associated with a better prognosis in UVM (Figure 1a). *RAF1*, a well-known proto-oncogene, is recognized for its capacity to activate the MAPK pathway, thereby contributing to critical cellular processes such as proliferation, apoptosis evasion, angiogenesis, genomic instability, and tumorigenesis [45,46,47,48,49,50,51]. Despite being consistently acknowledged as an activator of tumorigenic pathways, limited insights into the tumor-suppressive functions of *RAF1* have been documented. Notably, a study on hepatocellular carcinoma (HCC) revealed a protective role of *RAF1*, demonstrating its downregulation in HCCs compared to normal hepatocytes. The absence of *RAF1* expression resulted in the upregulation of YAP1, GP130, and STAT3 phosphorylation, activating the IL-6 family of inflammatory cytokines and leading to increased *CCL2* expression. Consequently, this process enhanced macrophage attraction [52]. Similarly, a study in urinary bladder cancer linked deletions in an *RAF1* region (3p25) with high tumor grades, advanced stages, and poor survival, which is consistent with our findings [53].

We also report findings on *SPP1* (osteopontin), a proto-oncogene that has been associated with inducing proliferation, migration, and macrophage attraction, among other effects [54]. Elevated *SPP1* expression has been reported in various cancers, including gastric, lung, pancreatic, colorectal, and cervical cancer, as well as in melanomas [55,56,57,58,59,60,61]. Interestingly, the subcellular localization of SPP1, whether in the cytoplasm or nucleus, leads to different survival outcomes. High cytoplasmic expression, measured by immunohistochemistry, shows a negative association with tumor grade, recurrent metastasis, tumor invasion, and recurrence, ultimately correlating with longer disease-free survival in colorectal carcinoma [62].

Moreover, *SPP1* is highly expressed in macrophages [63]. Analysis of single-cell RNA-seq data from uveal melanoma patients indicated elevated expression of *SPP1* in pro-angiogenic macrophages and inflammatory and antigen-presenting CAFs (Figure 7 and Supplementary Figure S2a). However, only a small subset of macrophages and CAFs expressed *SPP1*, suggesting a diverse role in UVM. According to a recent study, utilizing the same single-cell dataset as our analysis, at least four different types of macrophages exist in UVM [64]. Remarkably, C4 macrophages were associated with poor survival in the TCGA cohort, with a modest overexpression of *SPP1* in this type of macrophage (fold change: 1.1, adjusted *p*-value: not significant) [64]. The presence of *SPP1*+ macrophages is associated with poor prognosis, and this has been identified in colorectal and pancreatic cancer [65,66]. Our enrichment analysis of cells expressing *RAF1* and *SPP1* suggests the activation of interferon alpha and gamma responses, the TNFα pathway, and IL2 signaling (Figure 3), indicating that these macrophages may not be C4 but might be a mixture of C1, C2, or C3 macrophages, all of which exhibit an upregulation of these pathways. Nonetheless, the precise role of these macrophages in UVM prognosis remains unclear [64].

Our gene set enrichment analysis, comparing expression in low- and high-risk UVM patient groups, showed significant enrichment of the EMT phenotype in patients with UVM (Figure 3). This enrichment not only highlights the pivotal role of EMT in UVM metastasis but also suggests that EMT programming could be a key factor in enhancing the metastatic potential of BAP1-mutated UVM cells [67]. The ability of these cells to disseminate from the primary tumor site and their acquired capacity for self-renewal, which is critical for clonal expansion at metastatic sites, underscores the nature of this transition. This mechanism’s significance is further emphasized by previously published works, which have identified a prognostic gene expression signature centered around EMT genes. Such findings indicate that EMT genes are overrepresented in patients with a high-risk prognosis of UVM, providing a reliable framework for predicting disease outcomes and tailoring precise therapeutic interventions [68]. The characterization of key EMT genes, specifically ZEB1, Twist1, and Snail1, within UVM cell lines has revealed significant insights into the molecular underpinnings of this disease. In fact, ZEB1 emerged as a critical player, exhibiting the highest protein levels across examined cell lines and a significant association with highly metastatic cell lines. Furthermore, the observed correlation between elevated Twist1 expression and adverse prognosis in a separate tumor cohort underscores the prognostic value of these EMT markers [69]. Importantly, the mentioned study demonstrated that the genetic downregulation of ZEB1 and Twist1 leads to a substantial decrease in invasion and growth in selected UVM cell lines, highlighting their indispensable roles in tumor progression. In addition, higher expression of MMP1 and MMP12 in patients with tumors post-treatment compared to tumor-free individuals (Supplementary Figure S1) underscores the importance of these markers in predicting treatment response and tumor invasiveness. RAF1 showed decreased expression in patients with tumors post-treatment, suggesting its relationship with treatment response.

To evaluate the potential role of the immune system in survival differences between high-risk and low-risk patients based on this novel signature, we applied a deconvolution algorithm to estimate the composition of immune cells within the UVM microenvironment (Figure 5). We observed a heightened infiltration of iDCs in UVM, a class of cells that has received limited attention in this specific context. Despite the acknowledged challenges in eliciting effective tumor immunity, there are reports suggesting that tumor cells may exploit iDCs to promote tumor progression, influenced by tumor-derived soluble factors such as vascular endothelial growth factor (VEGF) [70]. The modulation of these iDCs within the tumor microenvironment highlights their dual role in both facilitating and inhibiting tumor progression, rendering them crucial targets for bolstering tumor immunity [71]. Our findings also support the involvement of M2 macrophages within the high-risk UVM group. This observation aligns with reports indicating that infiltrating macrophages in UVM predominantly exhibit an M2 phenotype, characterized by CD68+ CD163+ markers. The correlation between the abundance of these cells and factors, such as monosomy 3, ciliary body involvement, and increased microvascular density, underscores the prognostic potential of these immune cells. Furthermore, the association between high expression of CD68+CD163+ and diminished survival outcomes highlights the importance of the tumor microenvironment in disease prognosis and therapy [72].

Enrichment analysis performed on both bulk RNA-seq and single-cell data showed shared pathways such as TNF-alpha, indicating a concordance between the two approaches. However, several pathways were different, highlighting EMT-, IL-6-, and ECM-related pathways for bulk RNA-seq, and hypoxia, glycolysis, and MTORC1, among others, in single cells. Both approaches revealed pathways related to progression and metastasis. One reason for this difference is that for bulk RNA-seq, the comparison groups were selected based on our ECM signature risk score, which means that these pathways are dysregulated in patients with high expression of MMP1, TIMP1, and MMP12 and low expression of RAF1 and SPP1. On the other hand, single-cell analysis was performed separately for cells with high expression of MMP1, TIMP1, and MMP12 and cells with high expression of RAF1 and SPP1 due to a lack of overlap between the expression of high-risk and low-risk genes across cell types. Bulk RNA-seq pathways explain the processes occurring in the tumor, and single-cell pathways elucidate a more specific landscape in specific cells.

The association between our risk score and SCNA, DNA methylation, mRNA, and lncRNA molecular subtypes (Supplementary Figure S4) demonstrates the ability of this signature to stratify patients. The significant difference between clusters 3 and 4 compared to 1 and 2 for SCNA clusters shows that this signature is related to metastasis and chromosomal aberrations, as expected for uveal melanoma [73].

### Limitations and Future Directions

The heterogeneity of the marker expression of our signature within various cell types (Figure 7 and Supplementary Figure S2) underscores the necessity of validating expression patterns identified through bulk RNA sequencing in single-cell RNA cohorts. Several studies have demonstrated that bulk RNA sequencing is susceptible to confounding effects from mixed cell populations [74,75]. Hence, single-cell experiments are often integrated with bulk RNA sequencing plus deconvolution analysis to corroborate findings and vice versa. It is important to note, however, that a limitation of our study lies in the reliance on bulk RNA sequencing for signature construction, as well as validation using microarray data. Thus, confirmation through single-cell experiments, albeit limited by the availability of only 11 tumor samples, may not fully capture the complexity of UVM as a whole. In addition, although the initial selection of 97 ECM-related genes was not arbitrary (being based on GeneCards inferred functionality scores and MatrixDB, as described in Materials and Methods), we acknowledge that some ADAM family members were excluded due to the score-based filtering process. To address this, we performed univariate Cox regression analysis using expression and survival data from the TCGA UVM cohort to evaluate the prognostic relevance of bona fide ADAM and ADAMTS family genes (Supplementary Table S2). Finally, given the limited availability of effective first-line treatments for uveal melanoma, we conducted a drug sensitivity analysis to identify FDA-approved or investigational drugs that might show differential efficacy in high-risk patients classified by our ECM-based gene expression signature. However, we emphasize that these findings require validation and encourage further research to explore potential therapeutic applications.

## 4. Materials and Methods

### 4.1. Data Resources

The UVM patient RNA sequencing data were obtained from The Cancer Genome Atlas (TCGA) database (https://portal.gdc.cancer.gov/, accessed on 8 January 2023). Gene expression quantification was conducted on high-throughput sequencing (HTSeq) data, with fragments per kilobase million with upper quartile normalization (FPKM-UQ) as the unit of measurement for the gene expression dataset. Subsequently, the expression values were subjected to transformation using the base 2 logarithm, adhering to the unit “log2 (fpkm-uq + 1)”. The FPKM-UQ unit integrates gene length and read counts from RNA sequencing data to estimate gene expression levels. Data from a total of 80 UVM patients were obtained, ensuring the availability of both expression and clinical information.

### 4.2. Gene Prioritization

To identify genes linked to ECM proteins, we employed a systematic approach. Initially, we accessed the GeneCards database (https://www.genecards.org/ (accessed on 13 February 2024)). to extract the top ECM-related genes (*n* = 97). These genes underwent curation and were prioritized based on their relevance scores, as determined through Elasticsearch scoring. This scoring system comprehensively evaluates the alignment between a document and a given query term (i.e., “Extracellular matrix”), incorporating factors such as term frequency and query boosting [76]. An additional manual curation was conducted by validating each protein in the Extracellular Matrix Interaction Database (MatrixDB) [77] to establish their relationship with ECM processes.

### 4.3. Prognostic Model for Extracellular Matrix Proteins 

Univariate Cox regression analysis was performed using the set of 97 ECM-related genes to identify potential prognostic markers in the UVM TCGA cohort (*n* = 80). The survival module within the Tumor Immune Estimation Resource (TIMER) tool facilitated this analysis [15]. Genes showing a significant correlation with UVM patient survival were then selected for inclusion in the final prognostic model. Subsequently, we employed a Lasso–Cox regression approach using the R package ‘*glmnet*’ (version 4.1-8, available at: https://cran.r-project.org/package=glmnet, accessed on 13 February 2024) to classify patients into risk groups based on their gene expression profiles. A five-fold cross-validation was used to determine the optimal value for the hyperparameter lambda, with the partial likelihood deviance serving as a measure of model error. Using the lambda value derived from this process, we identified a subset of genes and calculated their corresponding coefficients to compute the risk score.

The risk score for each patient was computed by multiplying the gene expression level of each gene from the defined model by its corresponding Lasso regression coefficient. Subsequently, patients were classified into low- and high-risk groups based on whether their risk score fell below or equaled/exceeded the median value, respectively. This approach allowed the stratification of 40 UVM patients into the high-risk group and the remaining 40 into the low-risk group. To evaluate the prognostic value of the established model, survival analysis was performed using the UCSC XENA browser, wherein patients were divided into high- and low-risk groups [15]. Receiver operating characteristic (ROC) curves were utilized to assess the model’s performance. Additionally, to validate these results, the prognostic efficacy of the ECM signature was examined in the GSE22138 cohort [78]. Coefficients derived from the Lasso–Cox regression model were used to estimate the risk score in this independent cohort, following the same methodology as in the training cohort. Patients were subsequently categorized into low- and high-risk groups based on the median risk score, and survival analysis was performed using metastasis-free survival (MFS) as the outcome measure.

### 4.4. Analysis of Clinicopathological Features in Relation to Risk Groups

To examine the potential correlation between the clinicopathological features of UVM and low-risk or high-risk groups, we analyzed frequency distributions based on clinical data from the 80 UVM patients. Statistical analysis for this assessment was conducted using the SPSS v21.0 software (IBM Corp., Armonk, NY, USA). To compare the gene expression levels between UVM patients with low risk scores and those with high risk scores, we performed a differential expression analysis using the UCSC XENA browser. The gene expression data were normalized using the log transformation method to ensure an appropriate data distribution for subsequent analysis. Differential expression analysis was conducted employing the limma-voom method, which incorporates precision weight estimation based on the observed mean–variance relationship in the data. A significance threshold of 0.05 was applied to identify genes showing a statistical difference in expression, with control for the false discovery rate (FDR). A log fold change threshold of 1.5 was set to identify genes with substantial alterations in expression levels between the two groups. Finally, we utilized the publicly accessible AMC R2 platform (https://hgserver1.amc.nl/cgi-bin/r2/main.cgi (accessed on 25 March 2024)) to explore potential associations between gene expression levels obtained from the genes in the final model and clinical traits within the cohort of 80 UVM patients from the TCGA dataset.

### 4.5. Functional Enrichment Analysis

Overexpressed genes in the high-risk group were used as input for the functional enrichment analysis. For this, we used the top 100 differentially expressed genes (DEGs) and conducted enrichment analyses based on gene ontology (GO) and the Kyoto Encyclopedia of Genes and Genomes (KEGG) within the Enrichr web tool (https://maayanlab.cloud/Enrichr/ (accessed on 4 April 2024)) [79]. The results of this analysis were then visualized using the ‘*SRplot*’ R package, which generated enrichment bubbles and volcano plots. Utilizing all hallmark gene sets from the Molecular Signatures Database (MSigDB), we conducted a gene set enrichment analysis (GSEA) [80]. Enrichment scores were computed to evaluate the overrepresentation of gene sets within the dataset. To establish the statistical significance of this enrichment score, permutation testing was employed, and the FDR was applied to control for multiple hypothesis testing.

### 4.6. Analysis of Immune Microenvironment Infiltration

To evaluate the infiltration of immune and stromal cells in each of the 80 UVM samples from the TCGA, we utilized the R package ‘*xCell*’ through the web server (https://xcell.ucsf.edu/ (accessed on 6 April 2025)) [81]. We investigated the differential expression of immune and stromal cell infiltration between high-risk and low-risk UVM patients, employing a significance threshold of *p* < 0.05. In addition, we performed a correlation analysis between the expression levels of the five genes selected by the Lasso–Cox model and the infiltration scores of immunosuppressive cell types estimated by xCell. Specifically, Spearman correlation coefficients were calculated for each gene–cell pair across the 80 TCGA UVM samples. Only statistically significant associations (*p* < 0.05) were considered for visualization in a heatmap displaying the strength and direction of the correlations.

### 4.7. Drug Sensitivity Analysis Using pRRophetic for High-Risk Uveal Melanoma Patients

To identify potential drug treatments for high-risk patients, we utilized the “pRRophetic” R package [82]. This package integrates gene expression data with drug sensitivity information derived from a large panel of cancer cell lines, which serve as a reference dataset. The pRRophetic package applies ridge regression to the training expression data, using variable genes as predictors to estimate drug sensitivity, quantified as the half-maximal inhibitory concentration (IC50). We used this approach to predict IC50 values for the 80 tumors in the UVM-TCGA dataset based on their RNA expression profiles. Subsequently, we compared the IC50 values between high- and low-risk groups using either a t-test or Wilcoxon test, depending on the distribution of the IC50 data.

### 4.8. Validation Cohort

Transcriptomic data obtained from a cohort of UVM patients (*n* = 63, GSE22138) [78] was utilized to validate our prognostic model. This dataset included molecular profiles derived from gene expression microarrays conducted on enucleated primary tumors. The analysis employed Affymetrix U133plus2 arrays to scrutinize the transcriptomes of the UVM patients.

### 4.9. Single-Cell Analysis

To further investigate our prognostic signature, we conducted an in-depth analysis using single-cell data accessible in the GEO repository (GSE139829 accession). This dataset comprises samples from 11 tumors obtained from patients, with three samples derived from metastatic tumors and the remaining from primary tumors. Expression matrices were retrieved from GEO and processed using the ‘*Seurat*’ R package version 4.3.0, available at: https://satijalab.org/seurat/, accessed on 4 March 2024 [83]. In the quality control step, only cells expressing more than 100 genes (n_features > 100) and fewer than 10% mitochondrial genes (percent.mt < 10) were retained for subsequent analysis. Cell identification was accomplished using the ‘*singleR*’ R package. Visualization of cells with labels assigned by *singleR* was conducted using uniform manifold approximation and projection (UMAP), as implemented in the *Seurat* package. The *Seurat* function “add module score” was utilized to identify cells expressing genes within the prognostic signature, enabling the classification of cells into high-risk and low-risk groups. Furthermore, an enrichment analysis was performed for the potential identification of the transcriptional state of these cells and to uncover potential mechanisms underlying the observed differential risk. This analysis was carried out using the ‘*HypeR*’ R package version 1.7.0, available at: https://github.com/montilab/hypeR, accessed on 6 March 2024 [84].

### 4.10. Statistical Analysis

The comparison of clinicopathological variables between the high-risk and low-risk UVM groups was conducted using the chi-square exact test. Normality assumptions for continuous data were assessed using the Kolmogorov–Smirnov test. Survival differences based on gene expression or between the low-risk and high-risk groups were evaluated using the logrank test. Disparities in immune and stromal cell infiltration between the high-risk and low-risk UVM groups were calculated using the Mann–Whitney U test. A *p*-value of <0.05 was considered statistically significant for all comparisons, employing either SPSS v21.0 (IBM Corp., Armonk, NY, USA) or R software (v4.0.2, https://www.r-project.org/, accessed on 3 March 2023).

### 4.11. Association Between the ECM Signature and Uveal Melanoma Molecular Subtypes

To evaluate the association between the ECM signature and previously reported molecular subtypes, we downloaded cluster information for 80 UVM-TCGA patients from Robertson et al. [73]. Four different molecular subtype classifications were used for this analysis: somatic copy number alterations (SCNAs), DNA methylation, mRNA, and lncRNA, which are described in detail elsewhere [73]. For each classification, we compared different clusters with a t-Student or Wilcoxon test, depending on the normality of the data. In addition, we performed linear regression with the ECM signature risk score as the outcome and each of the clusters as an independent variable, taking the first cluster as a reference (the one with a better prognosis) using the lm ( ) R function from base R, version 4.3.1 (R Core Team), accessed on 8 April 2024, https://www.r-project.org/.

## 5. Conclusions

In this study, we developed and validated an ECM gene expression signature for prognostic prediction in patients with UVM. Our findings demonstrate that the ECM signature effectively stratifies patients into high- and low-risk groups, with significant differences in survival outcomes. The integration of bulk RNA-seq and single-cell RNA-seq data further highlighted the involvement of specific ECM-related genes and immune cell infiltration in tumor progression. This ECM signature holds promise as a potential tool for improving risk stratification in UVM, which could inform personalized treatment strategies.

## Figures and Tables

**Table 1:** Baseline clinicopathological data and correlations between risk score signatures.

Variable	All (*n* = 80) (%)	Low Risk (*n* = 40) (%)	High Risk (*n* = 40) (%)	*p*-Value
**Histological type**				
Epithelioid Cell	13 (16.2)	2 (5.0)	11 (27.5)	**0.002**
Epithelioid Cell|Spindle Cell	21 (26.2)	7 (17.5)	14 (35.0)	
Spindle Cell	30 (37.5)	22 (55.0)	8 (20.0)	
Spindle Cell|Epithelioid Cell	16 (20.0)	9 (22.5)	7 (17.5)	
**New neoplasm event occurrence—anatomic site**				
Liver	9 (11.2)	2 (5.0)	7 (17.5)	
Other, specify	5 (6.2)	4 (10.0)	1 (2.5)	**0.036**
Missing data	66 (82.5)	34 (85.0)	32 (80.0)	
**New neoplasm event type**				
Distant Metastasis	14 (17.5)	3 (7.5)	11 (27.5)	**0.050**
Locoregional Recurrence	2 (2.5)	2 (5.0)	0 (0)	
New Primary Tumor	3 (3.75)	2 (5.0)	1 (2.5)	
Missing data	61 (16.2)	33 (82.5)	28 (70.0)	
**New tumor event after initial treatment**				
No	59 (73.7)	37 (92.5)	22 (55.0)	**0.001**
Yes	20 (25.0)	3 (7.5)	17 (42.5)	
Missing data	1 (1.25)	0 (0)	1 (2.5)	
**Person’s neoplasm cancer status**				
Tumor Free	54 (67.5)	34 (85.0)	20 (50.0)	**0.002**
With Tumor	26 (32.5)	6 (15.0)	20 (50.0)	

*p*-values represent chi-square tests across full categories, excluding missing data. Significant differences (*p* < 0.05) are bold.

## Data Availability

The UVM patient RNA sequencing data can be accessed from The Cancer Genome Atlas (TCGA) database (https://portal.gdc.cancer.gov/, accessed on 8 January 2023). The single-cell RNA seq data used are available in the GEO repository under the GSE139829 accession.

## Supplementary Materials

The following supporting information can be downloaded at: https://www.mdpi.com/article/10.3390/ijms26094317/s1.
