# Correction: Mejía-García et al. Development and Validation of an Extracellular Matrix Gene Expression Signature for Prognostic Prediction in Patients with Uveal Melanoma. *Int. J. Mol. Sci.* 2025, *26*, 4317

**DOI:** 10.3390/ijms262412048

**Published:** 2025-12-15

**Authors:** Alejandro Mejía-García, Carlos A. Orozco, Julius Herzog, Oscar Alarcón-Betancourth, Alexandra Meneses-Torres, Marcela Ramírez, Johanna González, Yina Zambrano, Alba Lucia Combita, Diego A. Bonilla, Seth Frietze

**Affiliations:** 1 Department of Human Genetics, McGill University, Montreal, QC H3A 0G4, Canada; alejandro.mejiagarcia@mail.mcgill.ca; 2 Cancer Biology Research Group, Instituto Nacional de Cancerología, Bogotá 110311, Colombia; corozco@cancer.gov.co (C.A.O.);; 3 Translational Research Group in Oncology, Instituto Nacional de Cancerología, Bogotá 110311, Colombia; 4 Department of Biomedical and Health Sciences, University of Vermont, 106 Carrigan Drive, 302 Rowell, Burlington, VT 04505, USA; 5 Master Program in Epidemiology, Fundación Universitaria del Área Andina, Bogotá 110311, Colombiaameneses17@estudiantes.areandina.edu.co (A.M.-T.);; 6 Health and Sport Sciences Research Group, School of Health and Sport Sciences, Fundación Universitaria del Área Andina, Bogotá 110311, Colombia; 7 Department of Microbiology, Universidad Nacional de Colombia, Bogotá 111311, Colombia; 8 Research Division, Dynamical Business & Science Society, DBSS International SAS, Bogotá 110311, Colombia; dabonilla@dbss.pro; 9 Hologenomiks Research Group, Department of Genetics, Physical Anthropology and Animal Physiology, University of the Basque Country (UPV/EHU), 48940 Leioa, Spain

In the original publication [1], one cited reference was retracted after publication. This reference was only used to provide general background on ECM-related signaling and did not affect any results or conclusions. The reference has been removed and replaced with a suitable updated citation [2]. A short mechanistic clause relying on the retracted citation was removed. A correction has been made to the Discussion, Paragraph 2: Corrected text:

“…plays a pivotal role in tumor growth and cell motility contributes to tumor progression through extracellular-matrix remodeling and modulation of immune-cell interactions [26]. Recent single-cell and spatial transcriptomic analyses have shown that MMP1-expressing malignant subsets are associated with enhanced metastatic potential and immunosuppressive microenvironments [26].”

The authors state that the scientific conclusions are unaffected. This correction was approved by the Academic Editor. The original publication has been updated.
