# Supplementary figures and images for: Development and Validation of an Extracellular Matrix Gene Expression Signature for Prognostic Prediction in Patients with Uveal Melanoma
Source: Int J Mol Sci. 2025 May 1;26(9):4317. doi: 10.3390/ijms26094317 (PMC12072621; doi:10.3390/ijms26094317)

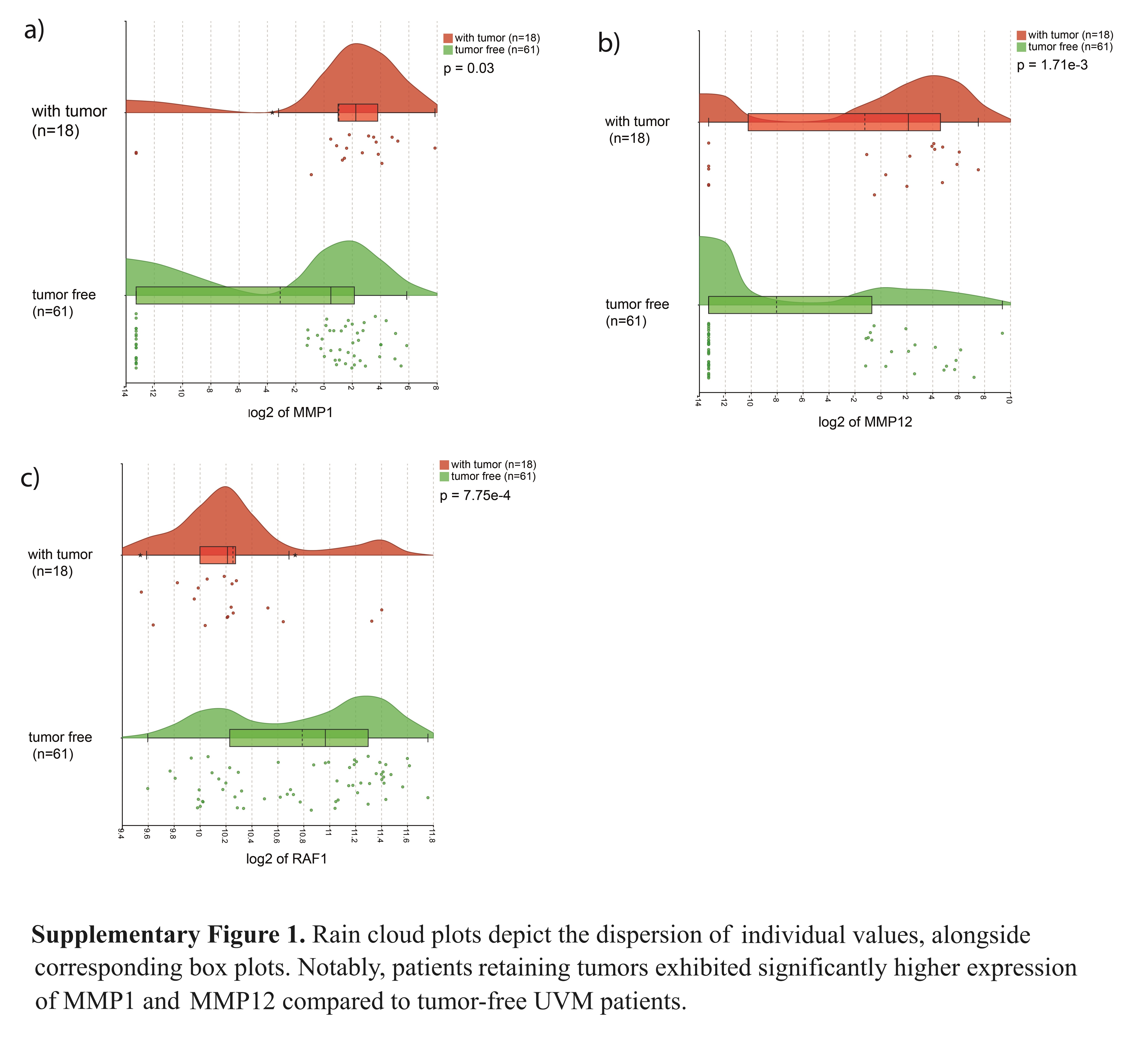

Supplement: Supplementary file 1 [file ijms-26-04317-s001.zip › ijms-3536340- supplementary/Supplementary Fig 1.jpg]

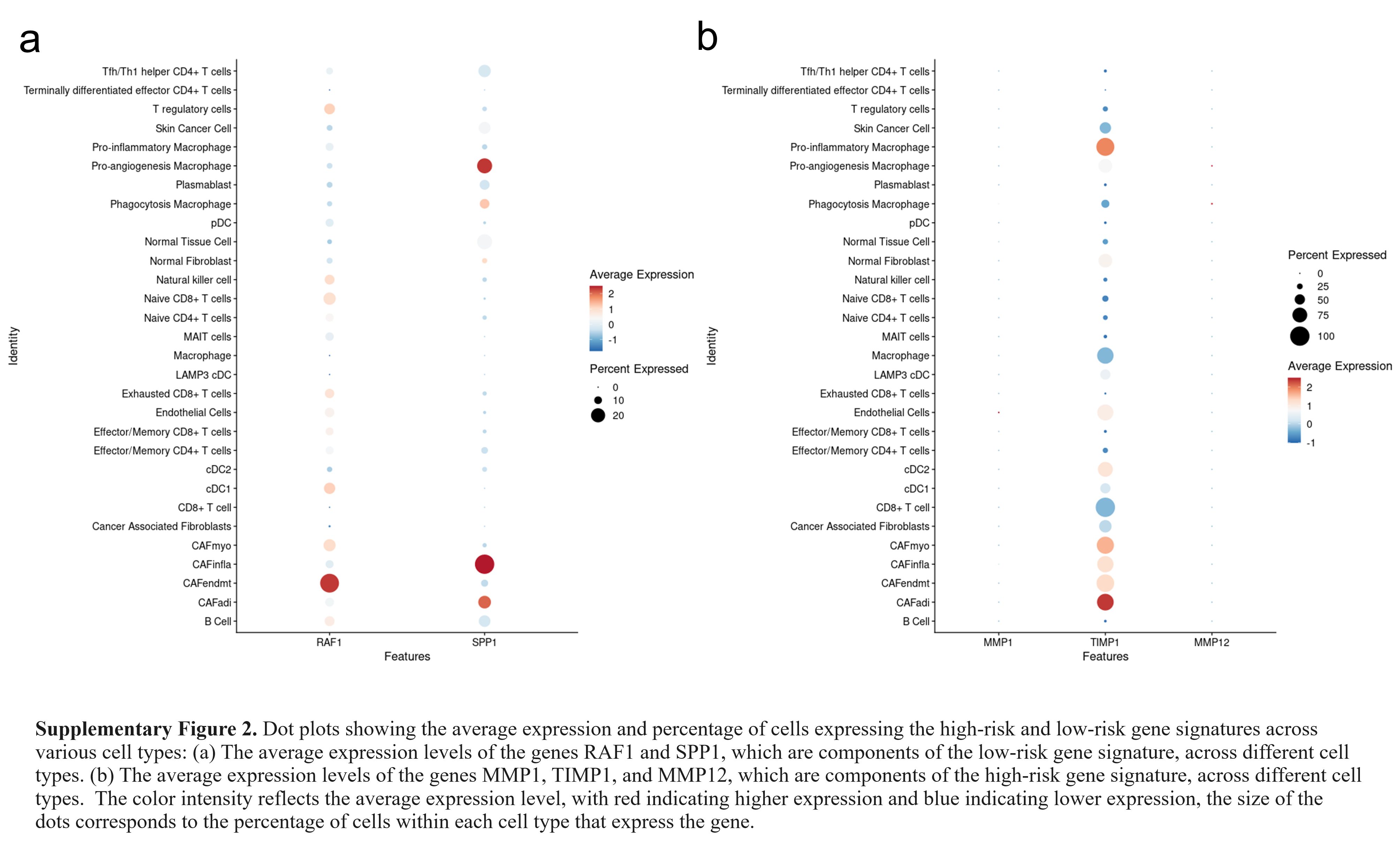

Supplement: Supplementary file 1 [file ijms-26-04317-s001.zip › ijms-3536340- supplementary/Supplementary figure 2 caption.tif]

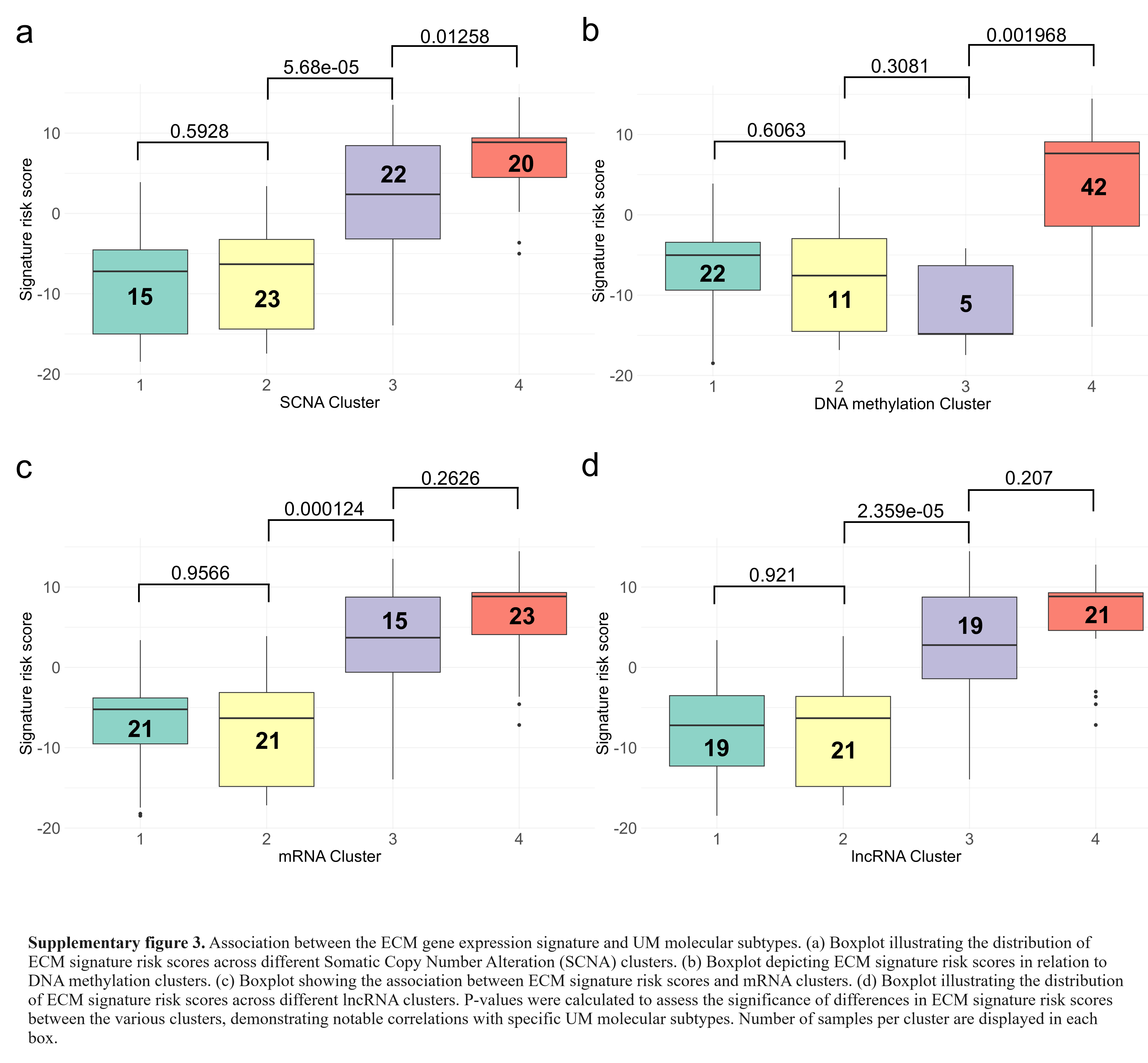

Supplement: Supplementary file 1 [file ijms-26-04317-s001.zip › ijms-3536340- supplementary/Supplementary figure 4 caption.tif]

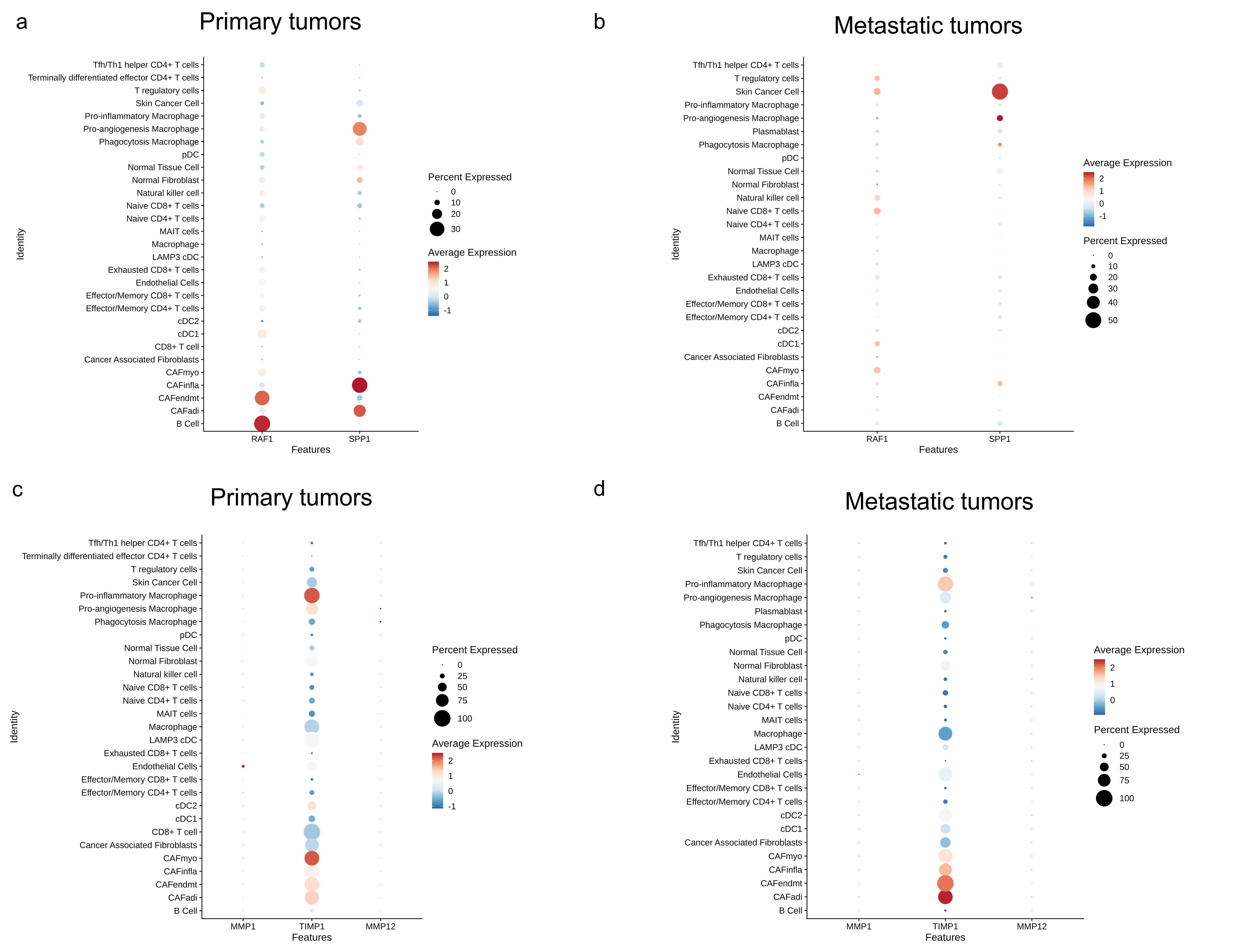

Supplement: Supplementary file 1 [file ijms-26-04317-s001.zip › ijms-3536340- supplementary/Supp_figure_3.tif]
